# A Review of Micro-Based Systemic Risk Research from Multiple Perspectives

**DOI:** 10.3390/e22070711

**Published:** 2020-06-27

**Authors:** Xiao Bai, Huaping Sun, Shibao Lu, Farhad Taghizadeh-Hesary

**Affiliations:** 1 Faculty of Economics and Management, East China Normal University, Shanghai 200062, China; baixiao@zufe.edu.cn; 2 School of Finance, Zhejiang University of Finance and Economics, Hangzhou 310018, China; 3 Institute of Industrial Economics, Jiangsu University, Zhenjiang 212013, China; 4 School of Public Administration, Zhejiang University of Finance and Economics, Hangzhou 310018, China; lushibao@zufe.edu.cn; 5 Social Science Research Institute, Tokai University, Hiratsuka-shi 259-1292, Kanagawa-ken, Japan

**Keywords:** systemic risk, method survey, systemic risk measures, complex network

## Abstract

The Covid-19 pandemic has brought about a heavy impact on the world economy, which arouses growing concerns about potential systemic risk, taking place in countries and regions. At this critical moment, it makes sense to interpret the systemic risk from the perspective of the financial crisis framework. By combing the latest research on systemic risks, we may arrive at some precautions relating to the current events. This literature review verifies the origin of systemic risk research. By comparing the retrieved and screened systemic literature with the relevant research on the financial crisis, more focus on the micro-foundations of systemic risk has been discovered. Besides, the measurement methods of systemic risks and the introduction of interdisciplinary methods have made the research in this field particularly active. This paper synthesizes the previous research conclusions to find the appropriate definition of systemic risk and combs the research literature of systemic risk from two lines: Firstly, conducting the division according to the sub-branch fields within the financial discipline and the relevant interdisciplinary research methods, which is helpful for scholars within and outside the discipline to have a more systematic understanding of the research in this field. Secondly predicting the research direction that can be expanded in this field.

## 1. Introduction

The study of systemic risk suddenly increased after the subprime crisis. Compared with the previous financial crisis, although there is an overlap between the two, systemic risk includes not only the information asymmetry, liquidity, and crisis transmission pathways involved in the financial crisis, but also the research on the stability problems caused by the structure of the system itself, the quantification of systemic risks, and early warning issues. As a result, further research on the market microstructure and agent heterogeneity has also been triggered. Meanwhile, cross-disciplinary research methods from other disciplines have been introduced, such as the introduction of complex network models when studying the structural stability of the system, linking the contagious effects of financial systemic risks to the transmission pathways of infectious diseases or bio-food chains [1,2,3,4,5,6], establishing new measures to measure systemic risk [7,8,9,10]. Different measurement methods in the financial sub-branch field are also applied to empirically verify and predict the tail loss of systemic risks [8,11,12].

What does systemic risk research study? What methods are used to study systemic risks? These are the questions that this review will always revolve around. The former one refers to a framework interpretation, which reveals the origin, predecessors, current status, and inner relationships between subdivisions. The methods are in service of the research purposes, thus the review of the later echoes the former one. This paper focuses on combing from two perspectives: first, from the perspective of the research subdivision of finance and economics, the traceability of systemic risk research can be clarified, and it is conducive to the theoretical research on financial crises and financial friction, which is very necessary. Given the natural link between systemic risk and financial crises, this paper adopts bibliometric analysis. Through analyzing the published papers in the two fields since 1992, we can know that the study of systemic risk and the study of the financial crises is in the same vein. Because systemic risk can be triggered by many factors, different market structures, participating entities, different transmission paths, and different impacts, there are many financial research branches involved. By sorting the number of citation index, we select the representative literature in the subdivisions and then conduct a literature review to grasp the focuses and main theoretical contributions of each branch. Such combing is also conducive to researchers outside the field of finance and economics research who want to follow the path of systemic risk. Another aspect is to sort out the methods in this field because it involves the intersections of sub-disciplines and interdisciplinary fields. The collected literature covers such disciplines as Finance, Accounting, Sociology, and Management. According to the keywords related to the method, recent core literature journals are thus collected and summarized. This review aims to provide a clear road map for the development of systemic risk research, linking traditional financial research with other disciplines. The current literature review of the methodology of systemic risk research is still limited [3] study is also a review of methodology, but does not summarize the sub-branches within the financial discipline). Besides, each method or theory has its advantages and disadvantages, and the problems that are suitable for solving vary. Thus, it is beneficial to obtain new methods or research conclusions by promoting research in the cross-field. This review will only be an attempt, subsequent studies and reviews will be expected to help further refine the research roadmap in this area. Therefore, the contribution of this paper lies in unblocking the path of systemic risk research within and outside the discipline of finance.

## 2. Progress in Systemic Risk Research

### 2.1. The Connotation of Systemic Risk

The scope of systemic risk and systematic risk is not the same. The latter refers to the risk factors shared by the entire economy, the non-distributable risk, also known as the market risk; it is opposite to non-systematic risk. Systemic risk refers to the risk of an overall collapse of the entire system due to the impact of individual member units, and systemic collapse is reflected in the impact of most or all individual members [13].

According to the Econlit economic literature database search results, the term “systemic risk” can be traced back to the speech of Andrew F. Brimmer, a Fed official in 1988, at a joint annual meeting of AEA (American Economic Association) and the Society of Government Economists in New York (This speech was published in the Journal of Economic Perspective the following year. See: [14]), there are different views on the clear definition of systemic risk in academia.

[14] first proposed the systemic risk and defined it as the occurrence and spread of a dilemma that is likely to seriously affect a country’s financial order. The dilemma itself may be derived from micro factors or macro factors. The BIS describes systemic risk as to the possibility that a party to a contract cannot perform an agreement and causes other contractors to default, and thus the chain reaction leads to a wider range of financial difficulties. This definition focuses on the overall risk of the system caused by the breach from microbodies. Besides, Darryl Hendricks [15] defines it from the perspective of equilibrium theory: systemic risk refers to the risk of moving the system from one equilibrium to another worse equilibrium, and this risk is difficult to reverse due to the catalysis of many self-reinforcing mechanisms. Following the systemic elaboration of systemic risks in 2003, [16] summarized the definitions of systemic risks and classified them into three categories: (1) The huge negative impact that the entire financial system faces at the same time, that is, systemic risks have the characteristics of simultaneity [17]; (2) All or a part of the financial institutions face the same risk exposure, which means systemic risks have the same cause [18]; (3)The impact can trigger a chain reaction. In other words, systemic risk is contagious [19]. [20] believe that these definitions have the following points in common: (1) Systemic risk concerns are for all financial institutions; (2) Systemic risk focuses on tail loss characteristics; (3) All need to consider the relevance and cross-cutting of the various institutions within the financial system.

Besides, there are differences and linkages between the concepts of systemic risk and financial crisis. [16] believes that systematicity is the most noteworthy cause of crisis formation and crisis development. The former is the reason and the characterization of the latter, that is, the financial crisis necessarily presents systemic risks, but systemic risks do not necessarily lead to financial crises. Moreover, current systemic risk research is mainly limited to financial systemic risk issues within an economy. The financial crisis has a wider scope and more diversified forms of expression. For example, depending on the scope of the spread, a financial crisis can be divided into bank crisis (the bank run), currency crisis, bubble crisis of capital market speculation, sovereign debt crisis, etc., and may trigger a comprehensive economic crisis. On the other hand, systemic risk is similar to financial risk in that it is contagious, and most or all financial institutions in the system will face the same impact at the same time. However, systemic risk emphasizes the impact of financial institutions on the overall risk of the system, that is, the endogenous nature of systemic risks, while the financial crisis may origin from endogenous or exogenous causes, for instance, for Iceland in the recent financial crisis, the US-centric financial crisis was transmitted to Iceland’s entire banking system, which led to the overall collapse of the country’s economy [21]. Besides, although systemic risks are not all triggered by the leverage (for example, on October 19, 1987, due to the stock market crash, the US stock index futures prices plummeted, causing the margin account to not add in time, leading to the collapse of the broker), the impact on the system after it occurs must be amplified with leverage. Therefore, the study of systemic risks is essentially related to the credit creation process and the degree of leverage. According to the different sectors created by credit, it can be divided into systemic risks of the banking system and the shadow banking system; according to the classification of financial markets and instruments that occur, it can be divided into systemic risks in the real estate market, banking system, bill market, securities market, bond market and derivatives market; according to the different debt departments, it can be divided into systemic risks of public debt (including systemic risks of sovereign debt), systemic risks of corporate debt, and systemic personal risks of households.

**Figure 1:**
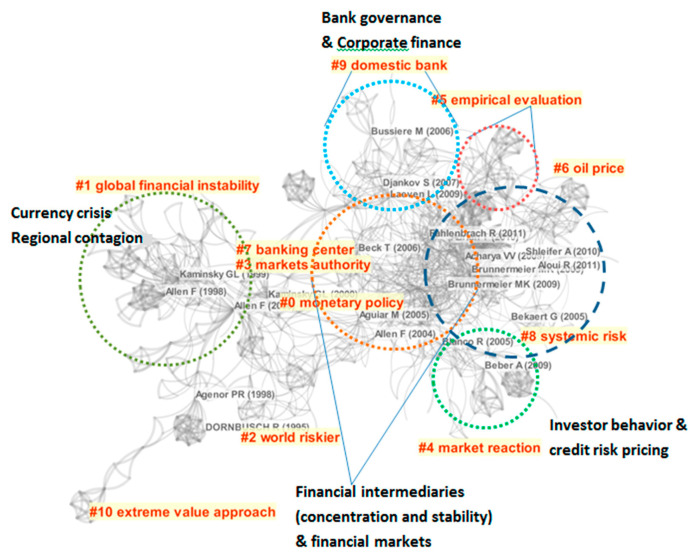
Overlapping of financial risk and systemic risk research. Source: Authors’ depiction from CiteSpace, 1025 papers are screened and analyzed according to their titles, keywords, abstracts and references. Among the 10 subdivisions, more co-citation clusters are formed in Regional contagion, Monetary policy, Credit risk pricing and Systemic risk.

On the other hand, because the financial crisis is highly correlated with the concept of systemic risk, the scope of the financial crisis and systemic risk research also has great overlap. By entering the keywords “financial crisis” and “risk”, a total of 7375 SSCI core documents and working papers published since 1992 were searched through Web of Science. By entering the keyword “systemic risk”, a total of 1913 core documents and working papers of the same period were searched. Through screening the titles and abstracts of all these papers by adding “financial markets”, 1025 papers are selected out. With the help of the software Citespace, further bibliometric analysis reveals that financial risk and systemic risk research have more crossovers, and the research concentrated areas are almost overlapping (Figure 1).

The sub-branches of financial crisis research are numbered according to the timeline of theory development. It demonstrates that early crisis research mainly focused on macroeconomic issues [22,23,24] As time went by, there was a detailed exploration into specific financial markets and product markets [25,26]. From another perspective, financial intermediaries and investor behaviors were discussed in terms of pricing credit risks [27,28]. As financial intermediaries have similar research methods with corporate finance, more later attention was drawn to bank governance [29]. As the Subprime Crisis erupted, more studies were oriented to the stability of the financial system, which overlaps with the systemic risk research [7,10,30,31,32]. A new branch, the extreme value approach, is also adopted in systemic research. Systemic risk research is not distinct from crisis research, as the former one also refers to such bank behaviors and liquidity problems. Among the 10 subdivisions, more co-citation clusters are formed in Regional contagion, Monetary policy, Credit risk pricing and Systemic risk. Within the Systemic risk field, the co-citation cluster of multidisciplinary research is the largest one, also with most focus on the financial network.

Thus, by combing the literature on financial crisis theory and systemic risk research, it can be found that the research on systemic risk is closer to the micro-level than the original financial crisis research, although systemic risk management involves macro-level management and supervision. There are more studies on the micro-market structure and participant behavior, and more emphasis on systemic risk transmission and contagious research, as well as the endogenous mechanism research that pays more attention to the occurrence of risks; it can be said that systemic risk research is the further deepening and refinement of the theoretical research on the financial crisis.

Therefore, although the academic community still has differences in the definition of systemic risks, by comparing the concepts of systemic risk and financial crisis, and summarizing the definition of systemic risk in the academic world, the concept of systemic risk can be defined from an economic perspective: triggered by macro or micro-events, the institutions in the system are subjected to negative impacts, and more organizations are involved in risk diffusion and the existence of internal correlations strengthens the feedback mechanism, causing the system as a whole to face the risk of collapse. The definition here emphasizes (1) Events triggering harms to institutions and systems; (2) Systemic risks that spillover and are contagious; (3) Due to the psychological and behavioral characteristics of the micro-subjects, a self-reinforcing feedback mechanism is formed; (4) The potentially serious consequence of systemic risk is the overall collapse.

### 2.2. Content and Category of Systemic Risk Research

**Figure 2:**
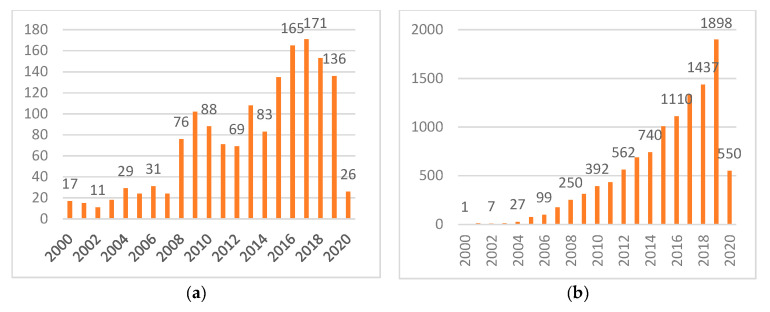
(**a**) Number of articles on systemic risk research published each year, (**b**) Number of articles on systemic risk research cited each year. Source: Web of Science.

We found that in Web of Science, from the Journal of Economics, Finance, Accounting, Behavioral Psychology, and Management, from 1992 to March 2020, all the articles on systemic risk published in the SSCI core journals totaled 1913 articles. From Figure 2a, we can tell the research on systemic risk has ups and downs from year to year, echoing with worldwide risk events. Besides, the search for working papers on systemic risk research on the NBER website has exceeded 200 publications in the past 10 years. Furthermore, the number of work papers published in this field newly released in 2020 is still considerable (Figure 2b). Especially, under current circumstances, systemic risk research deserves further exploring, as the Covid-19 pandemic struck the real economy by threatening human life first, then financial systems are impacted in terms of both assets and liabilities, which may further hurt real economies through systemic risk chains. Another peak in this area can henceforth be expected.

**Figure 3:**
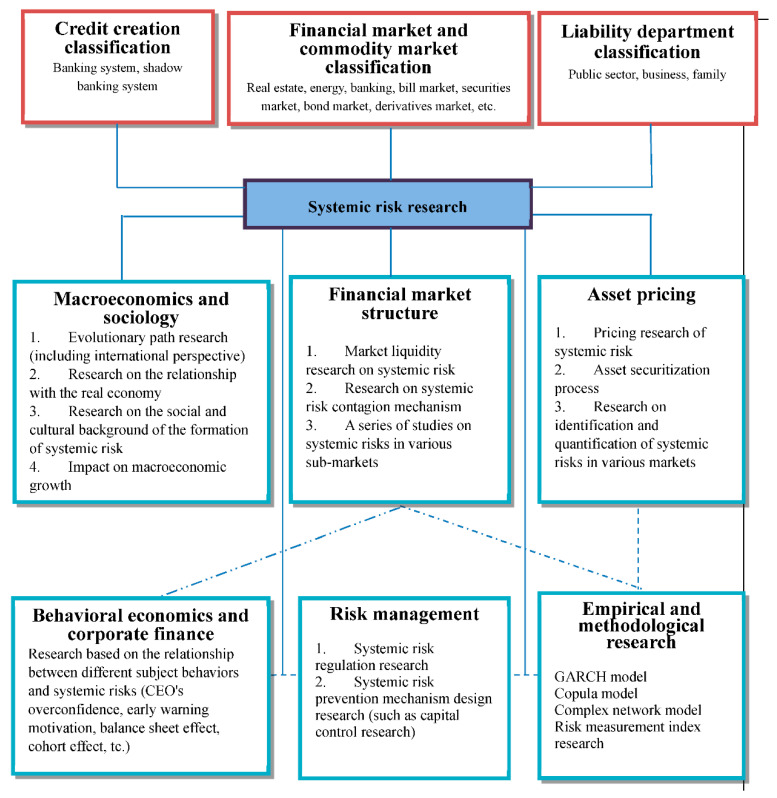
Sub-branches of financial disciplines involved in the systemic risk study. Source: Authors’ depiction.

According to [33] on the methodology of systemic research, the difference between systemic risk research and systemic risk lies in the unity of the whole and individual research. In addition to the macro and micro level research, it also focuses on the conceptual elaboration and model construction of different subject positions, as well as the analysis of internal and external relationships. To systematically sort out the previous research literature, this review screened out 100 articles based on the published journal grade, the total number of citations, annual citations cited, and the year of publication. According to the research content of these papers, it can be divided into evolutionary research on systemic risk formation, identification and measurement of systemic risk, and other empirical research related to systemic risk. Divided from the traditional financial research field, the above categories relate to different financial sub-areas: the evolutionary mechanism of systemic risk formation involves the study of market microstructure and behavioural subjects. Systemic risk identification and measurement involve asset pricing research. Other empirical studies related to systemic risk involve research on financial instruments, trading systems, financial regulation, and welfare economics. A tracing process can provide an overview of the general context of systemic risk research and facilitate the tracking of subsequent research (see Figure 3).

### 2.3. Research on the Evolution Mechanism of Systemic Risk

#### 2.3.1. Research on the Formation Mechanism of Systemic Risk

The research literature on systemic risk is sometimes the overlap of the fine branches of the Section 2.2 above. For example, the study of tail risk identification and quantification will involve specific empirical methods and recommendations including risk management. Therefore, the literature review in this chapter is mainly summarized and reviewed from the following four aspects:

The concept of systemic risk appears late, so systemic research on it is still being explored. However, according to the previous literature comparison, it has many similarities with the research theme of the financial crisis. The study of systemic risk formation mechanisms also draws on the research results of the formation mechanism of the financial crisis. Therefore, in the study of the formation mechanism of systemic risk, the research context of the formation mechanism of the financial crisis is first outlined.

The study on the formation mechanism of the financial crisis is mainly divided into two categories, namely, caused by emergencies or related to the economic cycle [34]. In the [35,36], bank runs come from depositors’ expected self-realization, which can explain some of the bank runs and the currency crisis, but it is difficult to use the model to make a prediction. [37] proposed that there is information asymmetry between banks and depositors. By establishing a global game model, the bank-runs problem is well explained, and the theoretical boundary value of bank runs is put forward. [38] extended this model to the mutual fund market and proposed an empirical approach to the global game model. The research of this branch is mainly related to the micro-market structure from the perspective of the micro-level and individual agent.

Another focus of research on the causes of the financial crisis is its procyclical nature. [39] pointed out that when the economy goes down, it is difficult for banks to realize the possibility of redemption. If depositors anticipate the impact of the economic downturn on the banking industry, they will withdraw funds from their banks, which may cause a run on the banks. [40] examined the economic volatility indicators of the United States from the end of the 19th century to the beginning of the 20th century and found that the leading indicators of the scale of debts of closed enterprises can accurately predict the occurrence of the banking crisis. [41], however, systematically studied the US comprehensive monetary policy from 1867 to 1960, and found that the four financial crises that occurred during this period were all caused by panic, but not related to the real economy. This led to a series of subsequent empirical tests on the causes of the financial crisis [42,43], which conducted a more detailed investigation of the four financial crises and collected more extensive data. It has been empirically found that the first three financial crises were triggered by the impact of the real economy, while the fourth financial crisis broke out due to panic.

The research on the systemic risk formation mechanism is carried out in the context of the establishment of the credible monetary system, and the degree of regulation of financial markets and the links between financial institutions and product innovations that have surpassed the previous era. Therefore, against this backdrop, the occurrence of systemic risk not only means that the liquidity of the bank or the banking industry itself causes a banking crisis but also means that the liquidity of the inter-bank market or the lending market in which it is involved in financing is exhausted. Furthermore, the resulting chain reaction, and even spread to other financial segment markets, eventually led to the collapse of the entire system. Therefore, the research on the formation mechanism of systemic risk is divided into two directions. The first one is the research on the formation mechanism of the financial crisis, which focuses on the micro-level; dynamic analysis is used to explore the effects of interactions within the system between individual behaviors, organizations, and financial markets. The other one is to focus on the institutional roots of systemic risk from the perspective of normative economics, or law and sociology.

[44] found that the frequency of financial crises in the world, such as the Great Depression, was, in recent decades, twice the sum of the number of financial crises in the Bretton Woods period (1945–1971) and the gold standard period (1880–1893). This cannot but make people wonder whether the shortcomings of the credit monetary system itself lead to the risk of a financial crisis, because the marginal cost of credit currency issuance is almost zero compared to its face value. Therefore, the central bank is more inclined to issue more than a limited amount of money to obtain money as a gain from credit capital investment or to achieve other policy objectives, especially when the central bank has a monopoly position and no other financial institution issues currency to compete with it. Moreover, the central bank is not bound by a bankruptcy (the self-owned capital ratio can be extremely low), so this tendency is more obvious. Besides, according to the guidelines proposed by the central bank theory founder Walter Bagehot that the central bank should follow (the loans provided by the central bank should set higher interest rates to prevent those who should not get loans, and the central bank should accept good bank collateral in the financial crisis to distinguish borrowers who are not solvent), this type of criterion, which can only rely on the central bank’s self-discipline, can easily be broken before or even during the financial crisis, as the Fed did in the 2007 subprime crisis. Therefore, [45] believe that the financial crisis is essentially a lack of restraint of central bank behavior, resulting in excessive credit funds, which is caused by an excess supply of high-quality assets. This view is consistent with the findings of [30,46,47,48,49,50,51], which showed that credit booms and asset price bubbles prevailed before the systemic banking crisis. [52] found that the financial crisis in many countries stemmed from the puncture of asset bubbles in the real estate market. As the financial crisis broke out, asset prices fell sharply and continued to have a major impact on real economic output and employment.

Further exploration of the deeper reasons, the premise of financial systemic risk accumulation as excessive debt, the root cause of excessive debt and what is the “degree” of liability, becomes an important part of the discussion of the formation mechanism of systemic risk. The discussion of this issue has even surpassed the scope of mainstream Western economic theory, so it is difficult to see the whole picture in the previous classic systemic risk research literature. George Bragues linked the act of capital interest-taking with the liberal democratic trend of thought, arguing that a large amount of lent funds had no tangible foundation beyond the credit creation ability of the banking system, and the fact that people exchange real goods and labor for this kind of currency without receiving any substantial return is unfair. It is the exploitation of the borrower by the lenders of the Greeks proposed by philosopher Aquinas. However, the idea of the rise of the liberal democratic notion that “everyone should be free to enter into a loan contract at an interest rate that is considered appropriate” justifies loan profitability. The function of credit creation in the banking system and the nature of profit-pursuing operation of capital will naturally lead to excessive debt of the overall economy, because “the main activity of finance is to create a huge debt chain and obtain more profits from it”. That is, due to the existence of compound interest, even if there is no new debt, the scale itself will have endless compound growth. The growth rate of the real economy is relatively limited, and the duration of high-speed growth is short-lived [53], which will inevitably lead to a continuous increase in leverage. Therefore, when debts accumulate to a certain scale, credit funds will inevitably spread more than high-quality assets, and due to the decentralized nature of globalization (financial centers and distribution centers in the West, product centers in the East, savings in the East, debt accumulation in the West, [54]), will become more dangerous, systemic risks and even the outbreak of financial crisis is inevitable.

Therefore, the free development of economy and capital may lead to disastrous consequences, requiring external supervision and constraints to restrict, and regulatory forbearance and legal loopholes are also important aspects of the study of systemic risk formation mechanisms. [55] believe that the outbreak of the subprime mortgage crisis stemmed from the SEC’s policy of exempting investment banks from restricting the regulation of the balance sheet liabilities in 2004, which was converted into an institutional voluntary supervision model, and the leverage ratio of investment banks generally rose sharply. The Commodity Futures Modernization Act, introduced in 2000, prevented state governments from using their laws to prevent Wall Street from using derivative financial instruments. [56] pointed out that relying on technocrats or over-reliance on technology to manage financial systemic risks will easily create an over-confidence illusion for regulators. As Greenspan admitted at the October 2008 congressional hearing, he relied too much upon and believed in the “self-correction of the free market.” Jonathan C. Lipson, a professor of law at Temple University, believes that the design and trading of financial derivatives CDS are divorced from the nature of their guarantees. Unregulated over-the-counter trading has made this market far larger than the actual guarantee, which has created hidden dangers for the outbreak of the subprime mortgage crisis.

Compared with the research on the formation mechanism of the financial crisis, the research on systemic risk formation mechanisms is not entangled in whether it is related to the real economy, but whether it is at the macro level or the micro level, whether it is institutional research or event research. On the other hand, research is more dispersed, and the formation mechanism of systemic risk spans the research scope of financial disciplines, which deserves more in-depth and detailed discussion.

#### 2.3.2. Research on the Impact of Systemic Risk on Economic Growth

[49,50,51] studied several financial crisis events from high-income and low-income countries, and found that the crisis would cause the average price of real estate to fall by 35% in the next six years; in the next three and a half years, asset prices fell by an average of 55%. In two years, real output fell by an average of 9%. In four years, the unemployment rate rose by 7%, while the government deficit averaged 86% higher than the pre-crisis level. Once the systemic risk evolves into a financial crisis, the impact on the real economy is obvious. As [57] put it, this is because external factors that are not related to the fundamentals of the company will lead to market participation shocks caused by changes in capital participation. This will affect the price of securities in the capital market, and thus change the investment behavior of micro-enterprises. The investment of enterprises has externalities and ultimately affects the operation of the real economy. He believes that different financing models have different impacts on the real economy. The equity financing market is suitable for investment projects that use equity financing methods (such as high-tech enterprises). Such enterprises have positive externalities to the real economy. Therefore, although the bursting of the technology bubble hit the securities market in the early 21st century, investment in high-tech industries has accelerated technological progress and is conducive to the development of the real economy. The bond financing market is suitable for investment projects that use debt financing (such as fixed assets investment such as real estate). Such investments are generally highly leveraged and have negative externalities to the real economy, so once the bubble bursts, the damage to the real economy is significant.

**Figure 4:**
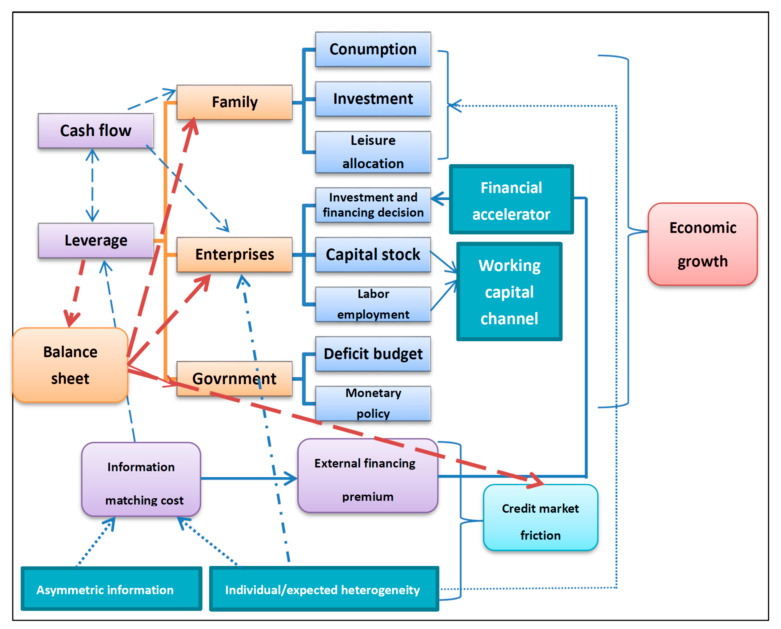
The transmission path of the impact of leveraged volatility on economic growth. Source: Authors’ depiction.

The theoretical study of the impact of systemic risk on the real economy has been dissociated from the mainstream of macroeconomic research for a long time after Keynes, because whether it considers from the real economic cycle model [58,59,60] or the Keynesian IS-LM model, the market is a complete market due to the alienation of individual heterogeneity or information asymmetry. Therefore, the financing method of enterprises does not affect the value of enterprises (MM theorem), so similar financial friction and systemic risk issues are difficult to match with the traditional theoretical framework, which makes the research on such problems lack micro-foundations. Until the new dynamic Keynesian theoretical framework (DNK) based on the DSGE method was put forward ((CEE) [61], (ACEL) [62]), it was compatible with the assumption of price stickiness, and incorporated the investment and financing behaviors and leverage changes under the information asymmetry into the model. The financial accelerator theory proposed by Bernanke et al., the BGG model [63], has become a pioneer in this field. Due to the limitation of the model’s solution, DSGE’s utility function and production function setting are both in the form of linear functions or quasi-linear functions. However, linear functions cannot explain the nonlinear changes brought about by economic shocks, and leverage has a nonlinear relationship with the economic cycle. The former has the most direct connection with systemic risk occurrence [64]. To explain the nonlinear relationship between leverage and economic cycles, Bernanke proposed a nonlinear investment and financing decision model linked to corporate net assets. In addition, because information asymmetry includes the cost of external investigations (costly state verification, CSV), there exists a risk premium between corporate internal financing and external financing (external financial premium). The existence of the premium will make the financing situation of the enterprise worsen when the economic shock occurs, which will accelerate the decrease in investment and bring about the “financial accelerator effect”. To simplify the research, Bernanke expressed the functional forms of other economic sectors as linear relationships, focusing only on the internal and external financing differences of enterprises and the resulting crisis transmission mechanism. Subsequent economists further extended financial frictions to the household sector, the banking system, and the government sector, and further explained the real estate market [65], the monetary policy transmission mechanism of government departments [66], and the risk transmission mechanism of the banking system [67,68] through the DNK theoretical framework. The transmission path that affects the real economy through leverage is not limited to the ways depicted in Figure 4.

The empirical study of the impact of systemic risk on the real economy is relatively infrequently referred only two of the top 100 works of literature in the annual average are related to this field. Even empirical studies of the impact of the financial crisis on the real economy are few and far between, which may stem from the following reasons: 1. The number of observable systemic risk events is limited, and fewer samples are analyzed using time series or panel analysis; 2. In the quantification of systemic risks due to the existence of many differences, the results of using quantitative indicators to assess the impact of systemic risks on the real economy may vary. 3. Because of the different ways of quantification, there will be large differences in the prediction of real economic fluctuations. If the results are not significant, the value of the research will be affected. [69] collected all systemic risk event samples after World War II in the United States and Europe to circumvent or mitigate the effects of the above problems, using 19 different methods to quantify and assess systemic risk. The quantile regression method was used to test the predictive power of systemic risk indicators on the deterioration of the real economy. It was found that only a small number of quantitative indicators can capture the risks of the macroeconomic downturn, and they can only be roughly predicted. Another reason for the lack of theoretical research on the impact of systemic risk on the real economy is that traditional macroeconomic theory itself has a weak micro-foundation, however, the systemic risk is mainly reflected in the systemic problems caused by the interaction of individual heterogeneous behaviours at the micro-level. By using traditional macroeconomic models, it is assumed that individual identity or limited heterogeneity has greater limitations. Therefore, [3] believe that the agent model (Agent model) should be introduced from other disciplines to better simulate the micro-individual behaviour, and to obtain a more realistic macroeconomic model.

Besides, the DNK model focuses more on the interpretation of risk-transfer pathways, and the endogenous interpretation of systemic risks is difficult. It is generally assumed that shocks are external, so it is difficult to predict the occurrence of crises.

As to the endogenesis, based on The General Theory, [70] proposed that capitalist economies had not shown to follow the neo-classical theory or in the “dis-equilibrium state” as Keynesian Economics stated, because of the functioning of sophisticated financial institutions. Two sets of prices, for current outputs and capital assets, aligned within a capitalist economy are determined by different proximate variables, as well as consumption demand and investment demand having different horizons, the former upon shorter run expectations and the later on longer ones. Both form the aggregate (effective) demand. Consumption demand is a function of current factors, while investment demand is a function of the price of capital assets, supply price of investment goods, expected profits and external financing conditions.

Different from the micro-based general equilibrium methodology, Minsky initiated his theory from a Macroeconomic perspective, based on Kalecki profit function, which always stands in a close or an open economy. By decomposition of the profit function, variables including monetary issues are demonstrated as follows:

π=I+DF−BP−sW+C

where π  is the current profit of the whole economy, *I* is the current investment of the private department, DF is the government deficit, sW is the savings of the private department, and C is the current consumption. Thus, profits are not only determined by current productive factors, government activities, savings and consumption, but also by asset prices, which connect closely with financial markets. Furthermore, profits, identified as cash flows, are the essential signals for investments, critically linking different payment commitments in the past, present and future. According to different cash flow commitments being availably financed, the financial system and debt structure are composed of a mixture of three types of financial postures (hedge finance, speculative finance and Ponzi finance). As the natural growth of the economy, profits, in the form of cash flows, fluctuate due to the endogenous discrepancy in expectation horizons, which causes the ratios of the speculative finance and Ponzi finance to increase in prosperous times, leaving the financial system to gradually become more sensitive to interest variations. When the current cash flows cannot sustain the current payment commitments, it initiates a deflationary break, and a financial crisis takes place. The implementation of mitigation policies can only alleviate the destruction yet not solve it, making the whole financial system more fragile to economic fluctuations. (Financial Instability Hypothesis).

Since the complication of interactions between key factors, as well as variant vast, complicates markets in capitalist economies, Minsky mainly demonstrated his theory in a narrative approach. Thus, even though Minsky’s theory became the kernel of post-Keynesian economics, it is difficult to further develop a comprehensive mathematical model ([71] once put Minsky’s FIH hypothesis into the framework of IS-LM model to explain and get the equilibrium result, but the conditional formula that equates the price of investment goods and the price of assets implies the assumption that the capital market and the commodity market will automatically maintain the equilibrium. Even though there are endogenous factors that cause deviation from the equilibrium of financial markets and commodity markets, the economy will return to equilibrium because of the implicitly assumed equality condition. Besides, the decision of asset price depends solely on investment demand, which is also contrary to Minsky’s view, who emphasizes that the determination of asset price depends on various factors.). It was also confronted with various criticism. [72] proposed that, as in the upwards phases of an economy, investment increases cause a higher level of aggregate profits so that the leverage ratio does not necessarily increase. While, in the downside phases, investment decreases negatively affect the aggregate profits more than expected, which will increase the leverage ratio. The paradox of debt goes against Minsky’s theory. This contradiction should be directed to the preconditions of FIH, which assume an extent of monopoly in commodity markets, so that producer can earn excess profits from market power. As the aggregate profits increase in the upward phases while the profit rates decrease, the over-increased investment would bring up leverage ratios, which could imperil future payment commitments. As in the downside phases, shrunk investments decrease aggregate profits while profit rates recover, which is good for bringing down leverage ratio. Thus, in the framework of Minsky’ theory, the paradox of debt is explainable on the presumption of monopoly markets. Further discussion can be referred to [73]. Some studies try to expand the Kaleckian model [74,75,76] by bringing a normal degree of capacity utilization into endogenous investment function. [77] utilize the profit share instead of the profit rate, [78] introduced external competition into an imperfect commodity market. All the adjustments provide a supplementary explanation of the demand regime. Furthermore, monetary and financial variables are incorporated into the profit function [79,80]. The late research intends to contain FIH and the paradox of debt through a Neo-Kaleckian model [81]. Moreover, by following a centre-periphery structure, the model explains how financial movements in the periphery associate with international financial markets’ movements and external vulnerability. Recent research in this field mainly focused on empirical research to support and verify FIH in different economies [82,83,84].

Minsky’s achievements are more than FIH. Instead, he focused on the whole system, aiming at constructing a general theory to explain the whole operational law of capitalist economies, including the upward and the downward phases of the economic cycle. The discrepancy in horizons and the amplification mechanism, which is realized through the interaction between profits and investment activities, as well as the positive (negative) feedbacks between asset prices and liabilities, not only explains the endogenesis of financial crises but also demonstrates his economic vision in a dialectical way, so whether the economy attains an equilibrium gives way to the depiction of economic operation processes, as the former is only a temporary stability status compared with the whole process. With regard to the difference in expectations on consumption demand and investment demand, it should not be criticized as a lack of rationality; it should be interpreted as generalized rationality, as in the “Wall Street view”, which is intended to be rationally applied to the real economy and sophisticated financial systems. 

### 2.4. Micro Market Structure Research on Systemic Risk

Early research on the formation of the financial crisis from the perspective of micro-market structure mainly began from the following two aspects: 1. Information asymmetry; 2. Liquidity. Besides, there are studies on the impact of the financial crisis from the perspective of currency and interest rates and expectations. Compared with the study on the financial crisis in the field of market microstructure, the study on the micro-market structure of systemic risk has increased the study of the complexity of the overall system, and it will be reviewed from the following three aspects:

#### 2.4.1. Research on Financial Network Complexity and Financial Stability

Before the outbreak of the subprime mortgage crisis in 2007, the conclusions of the close relationship between institutions and the complex financial network’s overall risk resilience were generally positive [22,85,86], and it was considered that sufficiently decentralized inter-bank liabilities could produce a more stable financial system. Nevertheless, some scholars then began to link the increase in systemic risk to the complexity of financial networks. For instance, [87] believes that as banks’ counterparties increase, the probability of a systemic crash increase. [88,89] demonstrated that the financial system itself has an amplification effect on the increase in systemic risk by modelling the inter-bank infection behavior. The research idea of this kind of amplification draws on the research methods of the previous network infection model [20,90], and the amplified transmission pathways are various. From assets and liabilities, studies by [91] showed that financially interrelated companies were more prone to systemic risk in the face of market shocks due to cross-shareholdings. Similarly, [92] argued that the similarity of assets held between banks determines the extent to which relevant information was disseminated and the likelihood of a systemic crisis. By studying bank exposures in the US and Europe, [93] argued that systemic risk was affected by common risk factors, and the interdependence of financial institutions was mainly due to systemic factors. [94,95] used asset securitization as an example to explain the transmission path of a financial network to amplify systemic risk from the perspective of debt. It is believed that through the interconnected balance sheet network, asset securitization essentially magnifies the financial leverage of the entire financial system, thereby increasing the vulnerability of the system. [96] showed that the higher the degree of financial integration, the more stable the interbank interest rate under normal conditions, and the higher the interest rate soars during the crisis. Combining the positive and negative views, [97] demonstrated through modelling that a closely linked financial system provides sufficient stability in the face of smaller market shocks. However, when the impact scale exceeds a certain threshold, the tightly connected financial network will amplify the risk and make the financial system more vulnerable, and the structure of the financial network has an important impact on the stability of the financial system.

The research of complex financial networks draws on the game model and the research results of the previous network theory. The network theory originated from the seven bridges problem and then developed into the random graph theory, and then evolved into complex network theory. Network theory and topological methods provided the basis for theoretical modelling of the structure of the research network itself [98] and also provided empirical methods [99]. Because how individuals interacting with each other in the network and their importance can significantly affect the results of network structure and impact transmission, the current research in this field is still very popular. [100] pioneered the use of an interbank debt matrix to describe the network structure of the banking system. This idea was followed by scholars who later studied systemic risk [97,101]. However, early network models used to be a non-direct model, which assumed that the connections between the participants are reciprocal and have the same interaction, even the same importance, such as if the inter-bank liability matrix is assumed to be positive, bank A’s claims against Bank B are the same as Bank B’s claims against Bank A. This is because the inter-bank liability data is not available (therefore, in the empirical case, the inter-bank liability behavior will be further assumed, and the banking system liability matrix will be determined through technical processing such as the information entropy method). On the other hand, for the sake of simplifying model simulation, as [97] described, the simplified model and the same subject are to study the relationship between the structure of the financial network itself and financial stability. The results of many empirical tests are contrary to the assumptions of the theoretical model. [102] studied the Italian interbank market structure from 1999 to 2010 and found that they are in line with the core-peripheral network structure, that is, a few banks continue to play a central role, maintaining lending with peripheral banks and providing liquidity to the market. While capital lending activities between core banks and non-core banks are rare, market liquidity at the time of the financial crisis is often caused by the reduction in borrowing by core banks, which means that the network distribution is not symmetrical. The core-peripheral network structure was first proposed by [103], other related works are contributed by [104], and [105,106] covered the most core-periphery network structures and a further supplementary version [107] was published in 2017. A series of empirical studies showed that in other financial markets, such as India, Mexico, the Netherlands and the UK, the banking system network also presented the characteristics of the core-peripheral structure [108,109,110]. Thus, more complicated networks with directional connections are introduced, which is more common in the financial contagion subdivision within the same E-N framework [111,112]. Moreover, since there are various types of connections between financial institutions, the financial network should have multi-layers, including credit, insurance, derivatives, collateral obligations cross-holding assets. The research on financial multi-layer networks is surging. [113] study the interaction of short- and long-term bilateral secured and unsecured lending. [114] study the Mexican banking system on all market layers and find that market-based systemic risk indicators systematically underestimate expected systemic losses.

#### 2.4.2. Research on Systemic Risk Related to the Behavior of Financial Market Participants

The research on the relationship between financial market participants’ behavior and systemic risk is more dispersed. The division of financial research segments can be mainly classified into behavioral finance research, corporate finance research, and micro-market structure research. Participants vary in different financial markets, the relationships between their behavior and systemic risk, and the transmission pathway, are also various.

According to the type of participant, the most studied is the bank. Despite the trend of bank disintermediation, in the financial crisis, the irreplaceable role of the banking system and its impact on the financial system determined that it is still at the core of the financial system. The idea that competition promotes the efficiency of financial allocation has promoted the wave of financial liberalization in developed countries in Europe and America since the 1970s (The second banking directive issued by the European Union in 1989 allowed banks to engage in banking, insurance and other financial operations; in 1999 the US Gramm–Leach–Bliley Act also eased similar restrictions.). Financial innovation products and means are constantly emerging, and competition among banks is becoming increasingly fierce, which brings about financial deepening [115] and improved resource allocation efficiency [116], but it may also lead to instability in the banking system [117]. The relationship between interbank competition and the stability of the banking system has always been controversial. On the one hand, competition is conducive to reducing the cost of capital, thereby increasing the return on investment of enterprises, and increasing profitability, which maintains the controllable credit risk of banks and increases the stability of the system [118]. On the other hand, it will lead to bank rent-seeking behavior, especially the bank deposit insurance system will make banks tend to take more radical measures to pursue profits in a fiercely competitive environment [119]. On this basis, without deliberately screening customers, the systemic risk will be increased [22,26,120]. This is because it is difficult for perfect competition to exist in the competition between banks. The different scales and core-peripheral banking system structure will inevitably have a monopolistic effect on this market [121], and there are moral hazards and adverse selection due to information asymmetry [122]. An extended study on the [118] models modifies the absolute inverse relationship between the two proposed [123,124], and presents that it should be U-shaped, that is, increased competition can improve the stability of the system and also make the banking system more vulnerable. The intensity of competition and other factors are the decisive factors that determine the negative or positive relationship between the two. [117] considered the role of policy incentives and constraints in the interaction between the two, and empirically tested and compared the relationship between the fierce competition in the banking industry and the stability of the banking system under different policy environments in different countries. It was found that the more standardized the regulation, the more developed the securities market, the more perfect the bank insurance system and the more efficient the credit information sharing, and that increased competition will increase the vulnerability of the system.

The method of balance sheet effect is not only adopted to analyze banking defaults, but also applied to study other market participants’ behavior. The fire sale and funding correspond to the activities of the bank’s assets and liabilities, respectively. When there is a run (trader’s run, deposit run or collateral run), the bank has to hedge the risk by recovering the loan in advance or raising the collateral mortgage rate, but there may be a risk of payment due to coordination failure. [34] believe that when the market lacks liquidity, banks have fewer opportunities to hedge their overall risk or deal with liquidity shocks. This will push banks to begin fire sales in response to future liquidity demands, which in turn will lead to excessive fluctuations in market capital prices. Moreover, when monopolistic behavior exists in the interbank market, it will further reduce the effective supply of funds in the market [125], and even market freeze appears.

#### 2.4.3. Systemic Risk and Market Liquidity Research

[30] classify liquidity risk into liquidity risk in the market and liquidity risk in trader financing. Traders provide liquidity to the market through financing, but the trader’s financing capabilities (such as equity and margin financing) rely on the liquidity of the asset market. However, market liquidity will suddenly dry up under certain circumstances and will affect many securities, resulting in large market fluctuations. It also leads to difficulties in the financing of traders and the realization of assets. The interaction between financing liquidity and market liquidity forms a liquidity spiral and triggers systemic risks. The liquidity spiral has a variety of transmission pathways, such as the loss spiral of erosion capital, and the guarantees spiral, which all lead to a fire sale. There are many different interpretations of the reasons for the formation of market liquidity shocks:

(1) Information asymmetry

Some of the research on financial crises caused by the lack of market liquidity is associated with information asymmetry, which is often one of the causes of liquidity exhaustion [122]. The financial crisis caused by information asymmetry is more typical in bank run events. The global game model proposed by [126] is also based on the assumption of information asymmetry, that is, participants can only obtain noisy observations, and the lack of common knowledge leads participants to choose risk-dominant equilibrium as the only equilibrium. This global game model is extended to the currency crisis model [127] and the bank crisis model [37]. When information asymmetry exists in the interbank market, it will exacerbate the liquidity of the market due to counterparty default risk, leading to system collapse [128]. Moreover, the information asymmetry between the supervisory authority and the regulated institution will cause the central bank to misjudge. Since regulators do not have a complete understanding of the asset quality of the entire banking system, therefore, even if a central bank participates in the interbank market to prevent the inefficient allocation of market resources due to adverse selection, moral hazard and monopolistic behaviour, the result of such hedging is difficult to be optimal [122].

(2) Liquidity mismatch

[129] believe that the common feature of financial markets in the recent financial crisis is that financial institutions generally have more serious mismatches in liquidity. For example, the terms of interest rate fluctuations in the subprime borrower’s loan contract will affect their ability to repay and refinance. Commercial banks are burdened with over-proportionate contingent liabilities relative to capital under unregulated off-balance sheet business. The rise in the proportion of investment banks that rely mainly on repurchasing commercial paper financing means that the proportion of the entire financial system that relies on market financing is rising, so the excessive leverage ratio is the risk of mismatch in liquidity. In the event of a liquidity shock, market liquidity is rapidly depleted by the balance sheet effect, resulting in the interaction of collateral run, bank run, and counterparty run. The central bank has to ease the crisis by releasing a large amount of liquidity, but this has further increased the moral hazard of financial institutions to increase leverage, which has further worsened the problem of liquidity mismatch.

(3) Actual demand shock

[130] argued that the demand for liquidity stems from the mismatch between the supply and demand of goods by consumers in different spaces. Once they needed to spend at different times in different locations, they constituted cross-regional liquidity needs, and banks needed to provide liquidity through the crediting behavior of the payment system or the interbank market. [22] agreed that random demand of consumers for liquidity will become a liquidity shock for a depository institution due to incomplete interbank market transmission. [86] suggested that systemic risk is mainly transmitted through the payment system, the interbank market and the derivatives market and that the random withdrawal demand of consumers at different times and in different places will have a liquidity impact on the payment system and the interbank market. The existence of the interbank market reduced the incentives for banks to hold non-profit cash; banks had solvency under certain conditions, while the market was caught in coordination failures.

The financial accelerator model proposed by [63] explained the external financing premium problem faced by enterprises from the perspective of the incomplete credit market. The incentive mechanism of the company has prompted the agent to favour debt financing. When the negative impact causes the net value of the enterprise to decrease, the solvency declines, which in turn has an impact on the asset side of the loan bank. When the assets of the loan bank are impaired due to corporate default and further affect the scale of the risk reserves, the bank faces a liquidity shock, and the impact has a nonlinear amplification effect through the interbank market.

Other factors affecting market liquidity risk were institutional and systemic risk management systems, financial system structures, and the institution’s own reputation risk [64]. Moreover, the phenomena associated with liquidity shocks include market freeze, asset sales, contagion effects, and institutional bankruptcy, which also possibly appear when systemic risks occur.

#### 2.4.4. Financial Contagions

Financial contagion occurs as the distress of one or a small group of banks risks the stability of other financial institutions, even ultimately spreading to the real economy. The financial contagion can occur due to both local contractual obligation connections, and global market connections, e.g., through the prices of assets due to mark-to-market valuation [131]. [100] established a framework for contagion analysis, which studies the spread of obligation default within the financial system due to unpaid liabilities. Since there are various types of connections between financial firms, the shocks can transmit in the network via different channels, and the channels also interact with each other in some historic events. According to [132] taxonomy, there are mainly four types of channels: asset correlated contagion, default contagion, liquidity contagion, market illiquidity and asset fire sales. Except for the default contagion, for the rest of the channels, the observed domino or spillover effects are demonstrated through the slump in mark-to-market prices. Thus, contagion research can be classified into two main branches: default contagion and price-mediated contagion.

(1) Default contagion

[133] explored how the probability and potential impact of contagion is influenced by aggregate and idiosyncratic shocks. They found that the robust-yet-fragile financial system has a low probability of such contagion. [134] extend the basic default contagion model by introducing default costs into the system. [135] assume minimal information about network structure, and through such key node-level quantities as asset size, leverage and the fraction of a financial institution’s liabilities held by other financial firms, they derive explicit bounds on the potential magnitude of network effects on contagion and loss amplification. Other models with extension in default costs include [90,136,137].

(2) Price-mediated contagion

These contagion models consider the mark-to-market asset price slumps due to the extreme tension in market liquidity and individual liquidity, as demonstrated in fire sales [138,139,140,141,142,143] cross-holdings have also been studied [91,136]. [144,145] provide a framework for modelling asset prices during a fire sale. [131] generalizes the model of [142] by allowing for differing liquidation strategies. [146] study the three extensions that are bankruptcy costs, multiple assets and fire sales within a single model.

The survey of empirical work on contagion can refer to [147]. Empirical studies are revealing that financial contagion can not be well explained by the base model of obligation default [101,135,145,148]. System stress tests are usually adopted to study the exposure of the whole system towards indirect contagion [138,149].

In general, the network-based models are mainly rooted in the general equilibrium approach, which brings sufficient economic connotation to these models. It also means that systemic shocks are usually assumed from outside instead of endogeneity. Thus, through limited amplification, contagions can suspend automatically and the whole system returns to a new equilibrium. Even referring to the multiple assets models, the shock on the prices of assets and consequent price variations distribute independently with the network contagion, otherwise, the mathematical model shall be too complicated to be solved. However, the financial contagion research does offer great achievements in:

(1) Explaining the contagion path via networks of all types;
(2) Empirically revealing the impact of contagion on the whole financial system;
(3) Providing supportive evidence for capital requirements, mark-to-market accounting and limitation of the scale of interbank business.

### 2.5. Systemic Risk Identification and Measurement

Systemic risk identification and measurement are one of the most important aspects of systemic risk research. In recent years, the literature in this field has also increased. The indicators of systemic risk are SES [7], MES [8], KLR [24], CoVaR [9] and Capital shortfall [10]. Some scholars have reversed the financial systemic risk through the market prices of derivatives (credit default swaps, CDSs) issued by financial institutions [150,151]. A survey of measurements on systemic risk can be referred to [152], and they also offered codes for 31 such measures.

Besides, [5] adopted the Herfindahl index to measure the concentration of credit portfolios of the emergency loans programs provided by the FED in the 2008–2010 crisis. They found that 22 strongly connected institutions received the most of the funds, while small dispersed shocks could have triggered the systemic default event, which indicates that the critical banks are “too-central-to-fail”. i [153] constructed an insurance pricing model based on the put option framework of the Merton model, including asset relevance, banking systemic risk and joint default rate, suggesting that systemic risk leads to low insurance pricing, which leads to insufficient liquidity in the event of a crisis. [154] used a multivariate extremum theory to model the tail dependence between multiple assets and adopted a non-parametric estimation to estimate and then to measure systemic risk. [155] studied the monthly yield data of hedge funds, banks, securities, insurance and other financial institutions based on principal component analysis combined with Granger causality, and concluded that the internal correlation of these four types of finance has increased the systemic risk. [156] viewed the banking system as a credit portfolio, analyzing the value of the portfolio and the unanticipated losses to calculate systemic risk. [157] considered financial departments as a time-varying systemic risk of overall measurements, and defined systemic risk as to the conditional default probability, and analyzed the default probability through credit repayment.

When the systemic risk is defined as a series of debt contract defaults, systemic risk is linked to credit risk. The biggest characteristic of credit risk is its tail effect. Therefore, many researchers have introduced the Copula model to simulate the tail dependence of financial institutions’ extreme losses under systemic risk [11,12]. [158] proposed a new systemic risk analysis framework, which incorporated marginal default probability, credit risk structure, consistent information multivariate density optimization (CIMDO), generalized dynamic factor model and t-Copula model into the framework. [149] propose the Endogenous Risk Index (ERI) to measure the spillovers across portfolios, as well as the Indirect Contagion Index (ICI) to capture the importance of a bank via measuring the loss it inflicts on other financial firms. The two indicators provide a complementary measure of interconnectedness. [159,160] provide reviews on more recent measurement studies. Therefore, it can be seen that, due to the tail characteristics and nonlinear characteristics of systemic risks, there are many methods for the quantitative assessment of systemic risks.

## 3. Review of the Main Research Methods of Systemic Risk

All approaches and definitions should serve their purposes. Due to the fast development of systemic risk research, this paper may not cover all the latest methods, while it can provide a series of frameworks for reference. According to the survey work, the approaches are classified into five categories: theoretical foundation, mathematical models, econometric methods, simulation and agent-based models. As to the simulation methods, more can be referred to [148], which will not be discussed in this section.

### 3.1. Theorectical Foundations

Systemic risk research rests its theoretic root in equilibrium approaches, which follow a micro-based research paradigm. To achieve the equilibrium, the route of deduction shall be clarified, and the micro-based equilibrium or activities need to be coordinated with the purpose of general equilibrium by a series of preset hypotheses. To explain the frictions on financial markets, the preset conditions are adjusted accordingly, based on more convincing and practical micro-foundations. There are at least three types of endogenous inconsistencies: inconsistency in preference or expectation [22,161], heterogeneity [22,26,86], information asymmetry [67,86], and BGG model [63]. Besides, through obligation contracts [22,26] or institutions’ capitals [86] or other generalized agreements [162,163], connections are established within the financial system, which establishes the network analysis of contagions, domino effects and spillovers.

As mentioned above, the study of the generation and transmission mechanism of systemic risk is in virtue of the DNK model framework. Linking the activity entities and market structure at the micro-level with macroeconomic growth and volatility makes the performance of macroeconomic fluctuation of systemic risk possess a certain micro foundation. Because systemic risk is accompanied by rising leverage, financial friction, large fluctuations before and after liquidity, and other derivative phenomena, it is closely related to individual heterogeneity and the characteristics of the market structure itself.

To explain the impact of individual heterogeneity or heterogeneous expectations on financial market asset prices and the real economy, Calvo and Rotemberg’s sticky-price model is used for reference. What’s similar is the application of the two-sector model in the utility sector or the constraint function in the family sector, for example, [65] distinguish the buyers from the superior and sub-optimal levels and separate their constraint functions, explaining the intrinsic relationship between the rapid rise of mortgage loans and the rapid rise in housing prices before the subprime mortgage crisis. The game model is also a more common method for explaining financial friction and systemic risk. [36] proposed an analytical approach to coordinated games. By dividing the depositor’s investment behaviour into two categories, patient and impatient, in the case of incomplete information, the investor will choose whether to withdraw the deposit to the bank in advance according to the expectation and then the bank run may occur due to the individual’s rational behaviour. This model successfully explains the reasons for the bank’s asset-liability maturity mismatch, and also explains the transmission mechanism that triggered the banking crisis. However, the deficiency is that there are multiple equilibriums in this model, and it is impossible to predict the occurrence of the bank run behaviour. The coordinated game model is then further optimized and expanded [37]. [126] proposed a method to ease public information hypotheses, allowing participants’ post hoc observations to satisfy certain random distribution characteristics. The game based on this hypothesis is named as a global game. Compared to the coordination model, the global game model eases the assumption of public knowledge and reduces the quantity of equilibrium. [127] introduced the global game method into the study of financial crisis theory and studied the mechanism of currency attack activities; the sequential global game method is also widely used in the fields of macroeconomics and financial crises. Chinese scholars also used this model to explain the liquidity crisis of banks and the government’s rescue policy [164], and infectious mechanisms for the liquidity of informal financial institutions to formal financial institutions [165].

As for the modelling of contagion and spillover risks among financial intermediaries, the covariance of assets [7] or liabilities [7,86,90] was introduced based on micro-level maximization, in which the agent is either risk-neutral or risk-averse. Asymmetric information assumption is also brought in.

Thus, the general equilibrium approach and its loose conditions can explain these endogenous questions: how participants react to an unexpected shock; what expectation they would form and action they would take thereafter; how the systemic risk is transmitted or spillovers occur within the system. However, although frictions are invited into the equilibrium models, they have to always assume the exogeneity of the shocks.

Another series of theoretical approaches are based on an analysis of evolutionary history and institutions, like Marxism and Minsky’s theory. The latter inherited the former’s essence in explanation of profits in that they both agree that profits go down as investment increases in the phases of economic expansion, and a crisis takes place when profits cannot cover the current payable obligations. While Minsky further developed his theory by establishing a profit function on an identical equation, it follows a macro-micro-macro non-equilibrium paradigm, which unravels the necessity of present assumptions. Through decomposition, the profit function can embrace various endogenous factors from different departments of an economy, including technical improvements, public policies and the financial institutions, etc., while Marxism mainly attributes the profits to the surplus-labour and capital structure. Minsky’s methodology can also be applied to analyze the household and government behaviours and its subsequent effects on the financial system. This methodology solves the problem of exogeneity, as the financial crisis phases are integrated into a whole theory to explain the operation of capitalist economies. To avoid mathematical formalism, he insisted on delivering his theory via a narrative approach, which leaves more space for his successors to explore [82,83,84], etc.

### 3.2. Mathematical Models

Network and contagion models are mainly established in the general equilibrium approach [166]. After [22], the baseline of the network model is proposed by [100], which is expanded from explaining default contagions to price-mediated contagions. Based on that, [133] offered a model involving hard defaults, in which interbank debts of defaulted banks recover zero value. There are also other models with extensions on bankruptcy costs, multi assets and fire sales.

[142] provides a framework to prove the existence of clearing asset prices and liability payments for equilibrium in a network contagion. [143] expand it to a dual-risky-asset equilibrium.

No matter the Eisenberg–Noe model, or the Gai–Kapadia model or the Amini framework, they all belong to static cascade models, which assume the structure and scale of the network itself be static. While networks can evolve due to the interactions of participants’ inter-connections of assets and shocks from the outside, so random graph models are brought in [6,132,140,142,167,168,169].

Moreover, to capture the stochastic and dynamic structure of the network with a vast volume of nodes, the mean-field models are borrowed from the discipline of Physics, which can be applied to explain the herding effect and endogenous contagion [170,171]. These models focus on quite simple interbank interactions that neglect defaults and contagion [137,170,172]. The latest models involve credit risk to explain default contagion [157,173,174].

### 3.3. Econometric Methods

Since, in most economies, the interaction data of financial institutions in the system is difficult to derive, it is also impossible to acquire the specific counterparty-trading information from a bank’s balance sheets. Thus, alternatives are adopted to settle this problem, one of which is the maximum entropy approach. The DebtRank approach is another measure to estimate the distress contagion without observing failing institutions [5].

The main methods used are the Copula model to simulate the joint distribution of risk factors, and the introduction of option pricing models and multivariate extreme value theory, or principal factor analysis to extract key factors using the DCC-GARCH model to model the correlation between multiple assets [8], and to establish multivariate quantitative indicators. Furthermore, to verify the economic structure variation as systemic risk takes place, VAR is usually adopted [82,83,84] tc.

### 3.4. Agent-Based Models

[175] proposed the introduction of an agent-based model (ABM) to solve individual heterogeneity problems, and Monte Carlo simulation can be used to predict the impact of micro-subject heterogeneity on macroeconomic variables. Compared with the game model, ABM is more compatible with more differences and can link the internal economic evolution logic with external data consistency. The key contributions by [176,177] are to incorporate the leverage accelerator into agent-based simulated network models. The limitation of such models is that it is difficult to calibrate and test their results.

### 3.5. Conclusions

In addition to financial heterogeneity issues, systemic risk draws more attention to the stability of the system itself, and complex network models are borrowed to solve such problems. Because of its similar structure to infectious diseases and bio-food chains, the financial system network links various financial institutions through financial flows, and triggers a domino effect due to risk events in individual institutions [22,97,178].

**Figure 5:**
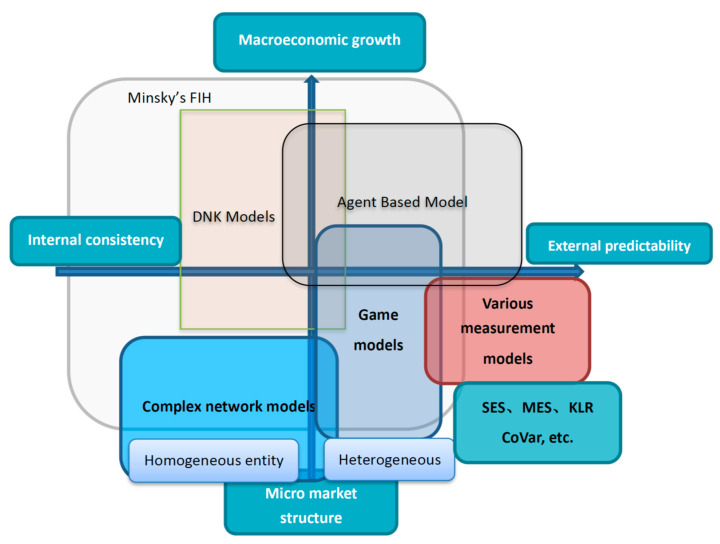
System diagram of systemic risk research method. Source: authors’ depiction.

However, the financial network system is more complicated than the natural food web or infectious disease transmission. This is because the activities of participating individuals are interactive, the interactions are not always symmetrical, the behavioral choices of the entity change with expectations or event shocks, and the results of behaviors are also characterized by uncertainty. This makes the financial system’s structural stability research more complicated and has to restrict the behavioral characteristics of the system to individuals through many strict preconditions [3]. The methods for identifying and quantifying systemic risks have been discussed previously. Therefore, in general, the system of research methods for systemic risk is as in Figure 5.

## 4. Future Expansion

Systemic risk is understood from the perspective of classical economics as the transformation between multiple economic equilibriums. From the perspective of risk management, it is tail risk management. From the perspective of behavioral finance, it is because the micro-subjects’ psychological and behavioral characteristics form a self-reinforcing feedback mechanism, which belongs to the systemic overall collapse risk from the perspective of complex network theory. From a broader perspective, it also covers the fields of sociology, psychology, and political science. There are a thousand Hamlets in the eyes of a thousand people. Different starting points focus on different problems, and the methods used are different. It is also the complexity and change of systemic risk that attracts researchers to constantly try to innovate. The systemic risk research branch is extensively involved, both in connection with traditional financial crisis research and expanding research on system structure and risk warning. Financial systemic risk research can be said to be the most intersecting field between finance and even economics and other disciplines. The main research methods used are both tools of traditional economic theory, as well as social network models and natural science experimental simulations. For the generation of systemic risks, the endogenous problems of shocks are difficult to solve using traditional economic models. Therefore, DNK’s DSGE model often assumes that shocks are exogenous; to predict systemic risks, it is urgent to introduce interdisciplinary approaches to innovate research on endogenous factors. On the other hand, the adoption of complex network models often requires many rigorous assumptions that are outside of reality, and these assumptions may exclude the potential risk factors of reality. After all, the financial system itself is much more complicated than the food chain, while other new interdisciplinary research methods also have insufficient internal economic explanatory power. It is, therefore, necessary to use appropriate analytical tools based on specific issues of the specific financial sector.

**Figure 6:**
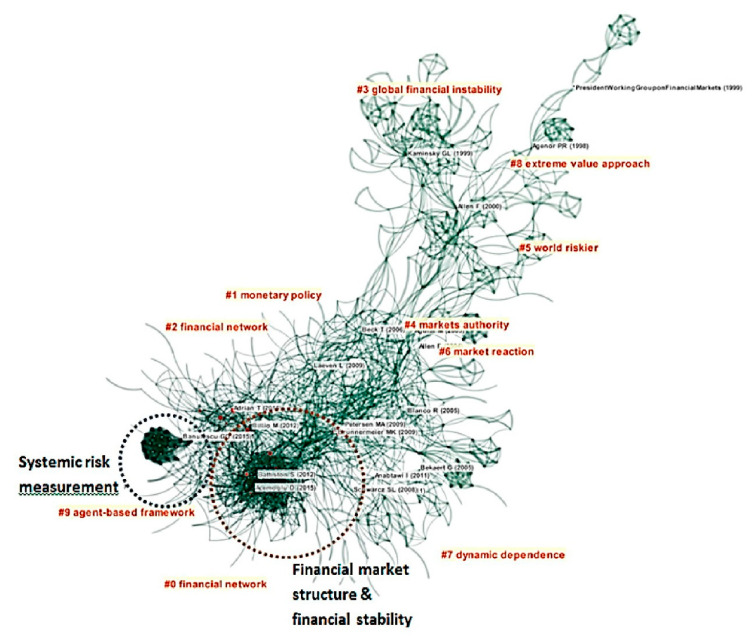
Latest systemic risk research map. Source: Authors’depictation from CiteScope.

To better sort out the hotspots, the latest 5 years’ (2015–2020) systemic risk research was analyzed and was connected with the development of crisis research, 733 articles in total. It turns out that two divisions are mainly focused on (Figure 6).

A total of 733 articles are screened and analyzed according to their titles, keywords, abstracts and references. Among the 10 subdivisions, more co-citation clusters are formed in systemic risk measurement, financial market structure, and financial stability.

This map reveals that more recent studies focus on network stabilization and systemic risk measurements. Although the extreme value approach was brought into this area, it caught only a small amount of attention. Furthermore, the high concentration on risk measurement may imply that the measuring methods gradually mature, which leaves limited room for subsequent researchers. Considering the ongoing worldwide recession, the research directions that may be further expanded in the future are as follows:

(1) More work on models with multiple shocks and spillover effects be done. No matter endogenous or exogenous shocks, the contagion rotation shall not be limited to a specific market or intermediaries. More than one spillover risk is discussed within a model. Moreover, at present, there are many types of research on systemic risks in the banking system, the real estate market and the foreign exchange market. There are few studies on the systemic risks of the shadow banking [179,180] and internet financial markets, which may be related to the lack of available data and difficulties in providing supporting empirical studies.
(2) Dynamic multi-layer networks expect further exploration, especially when the prices of assets have a connection with the financial contagions.
(3) More empirical studies of the impact of systemic risk on the real economy are necessary. It is expected that recent advances in large-scale data and computing tools benefit systemic risk studies, acquiring more real-world and large-scale data for empirical analysis [181].
(4) Event studies shall be focused on the modification of market structure, which is being affected by new technology or policies that help promote information disclosure, for example, the alternative index (like SOFR) in place of LIBOR. Moreover, digital currency issued by central banks substituting banknotes means that the monetary multiplier is no longer applicable, which will profoundly alter the whole financial system. Therefore, these innovative measures should also be paid attention to.
(5) Systemic risk analysis of China’s financial market. The regulation of China’s financial market is just like modular management, which can cut off the transmission of systemic risks in a special period. Coupled with the intervention of government funds, there has been no substantial systemic collapse in China’s financial markets. Therefore, it is necessary to study the systemic risk of China’s financial market, and study what effective measures mitigate systemic risks and whether the trade-offs between the costs and benefits of these measures are optimal.

Besides, there are other areas worth exploring, such as more comprehensive risk warning indicators, more effective econometric means, and crossover with other fields of economics or sociology, psychology, etc. In short, this is an active research field and has a strong practical significance and policy reference value. The research of systemic risk will be more vibrant because of the participation of more researchers.
